# Knocked-Out *Bombyx mori* Protein Disulfide Isomerase Decreases Silk Yields and Mechanical Properties by Affecting the Post-Translational Modification of Silk Proteins

**DOI:** 10.3390/insects16070684

**Published:** 2025-06-30

**Authors:** Shifeng Yang, Mengyao He, Xian Li, Huan Dong, Hexu Lei, Fangyu Wang, Hanxin Deng, Hongji Zhou, Siyu Chen, Yujuan Zhou, Zihan Meng, Ding Tu, He Wang, Qingyou Xia, Feng Wang

**Affiliations:** Integrative Science Center of Germplasm Creation in Western China (CHONGQING) Science City, Biological Science Research Center, Southwest University, Chongqing 400715, China

**Keywords:** *Bombyx mori*, *BmPDI*, CRISPR/Cas9, silk gland, silk properties

## Abstract

Silkworms synthesize and secrete silk to produce cocoons, which are excellent materials for textile and biomaterial manufacturing applications. However, the gene regulation associated with the post-translational modification of silk proteins remains unknown. In this study, we analyzed the physicochemical properties, evolutionary relationships, and expression patterns of *BmPDI* in silkworms. Subsequently, we knocked out *BmPDI* (*BmPDI*-KO), resulting in significant phenotypes of *BmPDI-KO* silkworms with smaller silk glands and cocoons, weaker silk mechanical properties, and reduced disulfide bonds in silk-associated proteins. Transcription levels of silk protein-related genes and unfolded protein response signal pathway-related genes were significantly downregulated. In contrast, genes involved in the apoptosis pathway were significantly upregulated in *BmPDI*-KO silkworms. Knocking out *BmPDI* in silkworms affected the post-translational modifications of the silk proteins, thereby accumulating misfolded silk proteins and hindering their secretion into the extracellular cells. This further increased endoplasmic reticulum stress, activated the apoptotic pathway, accelerated silk gland cell apoptosis, and significantly reduced the silk yields and mechanical properties of *BmPDI*-KO silkworm. This study provides a potential exploration of *BmPDI* in the modification of silk yields and mechanical properties of *Bombyx mori*.

## Simple Summary

This study utilized CRISPR/Cas9 gene editing technology to knock out the *Bombyx mori* protein disulfide isomerase (*BmPDI*) at the individual level of silkworms, and comprehensively applied molecular biology, material mechanics, and other techniques for related detection. The preliminary results showed that the *BmPDI* gene might affect silk yields and mechanical properties by regulating post-translational modifications of silk proteins.

## 1. Introduction

The silkworm (*Bombyx mori*) is a typical *Lepidoptera* family representative and an economically important insect because of its ability to synthesize and spin large amounts of silk to make cocoons. The silk gland is the only organ that produces silk proteins in the silkworm and can be morphologically divided into three parts: the anterior (ASG), middle (MSG), and posterior silk glands (PSG) [1]. Fibroins and sericins are the two primary components of silk proteins; fibroins are synthesized in PSG, and sericins are synthesized in MSG [2]. Fibroins comprise a 350 kDa fibroin heavy chain (Fib-H), a 26 kDa fibroin light chain (Fib-L), and P25 of approximately 30 kDa [3]. Disulfide bonds link Fib-H and Fib-L to form complexes and are combined with P25 in a 6:6:1 molar ratio to form an elementary unit of fibroin [3,4]. Sericins primarily comprise *Sericin1* (*Ser1*), *Sericin2* (*Ser2*), and *Sericin3* (*Ser3*), which bond two silk threads together [5,6].

Silk proteins exhibit biocompatibility, morphological plasticity, and biodegradability, making them considerably valuable in composite materials, regenerative medicine, military products, and beauty products [7,8,9]. In biomedicine, fibroin proteins can be used in surgical sutures, artificial skin, drug stents, aerospace materials, and nanocomposite materials [10,11,12]. In medical cosmetology, sericin has various biological activities such as whitening, anti-radiation, anti-oxidation, maintenance of cell proliferation, and lowering of blood pressure [13,14,15,16]; thus, it can be used to produce cell media and cosmetic additives. However, the improvement of silk properties and performance through genetic modification remains challenging.

Protein disulfide isomerase (PDI), a member of the thioredoxin (TRX) superfamily, is a highly abundant protein mainly located in the inner lumen of the endoplasmic reticulum (ER), with a TRX-like structural domain active site [17]. The classical PDI protein comprises four TRX-like structural domains, a, b, b′, and a′ with an exceedingly acidic C-terminal extension c and a b′-a′ linker x, forming the a-b-b′-x-a′-c protein structure pattern [18]. The function of PDI in ER participation is to catalyze the formation of protein disulfide bonds and protein folding, correct the isomerization or rearrangement of misfolded disulfide bonds, and act as a molecular chaperone to inhibit the aggregation and precipitation of misfolded proteins [17,18,19]. PDI is closely related to the occurrence of diabetes, Alzheimer’s disease, Parkinson’s disease, tumors, and some cardiovascular diseases [19,20]. Mutations in PDIA3 (also known as ERp57), a member of the PDI protein family, lead to neurodevelopmental defects in zebrafish, such as axonal disorganization and skeletal abnormalities [21].

In silkworms, *Bombyx mori* protein disulfide isomerase (*BmPDI*) exhibits a high expression level in the fat body and silk gland and can be regulated by ER stress, hormonal regulation, and exogenous bacterial infection [22]. However, the role of the *BmPDI* in silkworms, particularly its effects on the post-translational modification of silk proteins, remains unclear. In this study, we systematically analyzed the physicochemical properties, evolutionary relationships, and expression patterns of *BmPDI*. We also investigated the role of *BmPDI* in regulating the development of silk glands, its effects on the synthesis and secretion of silk proteins in silkworms, and the structural properties of silk fibers. This study provides potential for further exploration of *BmPDI* in silk genetic modifications.

## 2. Materials and Methods

### 2.1. Silkworm Rearing and Sample Collection

The silkworms (*Bombyx mori*) used in this study were provided by the Biological Science Research Center at Southwest University, China. Larvae were maintained under standard rearing conditions at 25 ± 1 °C and 75% relative humidity, fed with fresh mulberry leaves. Tissue samples, including the head, epidermis, midgut, silk gland, fat body, malpighian tubules, testes, and ovaries, were dissected from fifth-instar larvae on day 3. Silk gland samples from fifth-instar larvae were collected daily (days 1–5) and categorized into anterior (ASG), middle (MSG), and posterior (PSG) regions. Additionally, PSG samples were obtained from multiple silkworm strains on day 5 of the fifth instar. All samples were stored at −80 °C until further analysis.

### 2.2. Bioinformatics Analysis

The cDNA (NCBI accession: NM_001043706.1) and protein (NP_001037171.1) sequences of *BmPDI* were retrieved from the NCBI database. Structural and sequence analyses were conducted using ExPASy (https://www.expasy.org/, accessed on 22 May 2022), SMART (http://smart.embl-heidelberg.de/, accessed on 27 May 2022), and ClustalX v1.8.

### 2.3. Quantitative Real-Time Polymerase Chain Reaction (qRT-PCR)

Total RNA was isolated using TRIzol reagent (Invitrogen, Waltham, MA, USA) following the manufacturer’s protocol. qRT-PCR was performed on a qTOWER2.2 instrument (Analytik Jena Biometra, Jena, Germany) with SYBR Premix Ex Taq (TaKaRa, Maebashi, Japan). Each 20 μL reaction contained 100 ng cDNA and 0.8 μM primers. Thermal cycling conditions were as follows: 95 °C for 30 s (initial denaturation); 40 cycles of 95 °C for 15 s, 60 °C for 30 s, and 72 °C for 30 s. The eukaryotic translation initiation factor 4A gene (*BmEIF4A*; SilkDB Probe sw22934, BGI, Chongqing, China) was used as the internal control. The relative expression level of the target gene was calculated using formula 2^−(Ct gene Ct control)^. Three biological replicates were analyzed. Primer sequences are listed in Table S1.

### 2.4. sgRNA Design and Vector Construction

Three sgRNAs targeting exon 1 of *BmPDI* were designed using the CRISPR/Cas9 Target Online Predictor (https://cctop.cos.uni-heidelberg.de:8043/, accessed on 12 June 2022.). These sgRNAs were cloned into the pBac[3×P3-EGFP-TTTTTT-U6] vector [23] to generate the final construct pBac[3×P3-EGFP-U6-*BmPDI*-gRNA].

### 2.5. Embryonic Microinjection and Screening

A 500 ng/μL mixture of pBac[3×P3-EGFP-U6-*BmPDI*-gRNA] and hsp70-PIG helper vector [24] was microinjected into silkworm embryos (0–2 h post-oviposition) using an Insect Embryo Micro-Injector (IEMJ, Chongqing, China). Injected G0 embryos were incubated at 25 °C and 85% humidity until hatching (~8–10 days) [25]. G0 adults were crossed with siblings or wild-type (WT) moths to produce G1 progeny. Positive G1 individuals were identified by EGFP fluorescence in embryonic eyes using a stereomicroscope (Olympus, Tokyo, Japan). Fluorescent G1 larvae were crossed with a Cas9-expressing transgenic line (Cas9^EXP^) [26] to generate F1 mutants.

### 2.6. Phenotypic and Economic Trait Analysis

F1 larvae co-expressing gRNA and Cas9 were screened via fluorescence microscopy. Genomic PCR confirmed *BmPDI* mutations. Silk glands from WT and *BmPDI*-KO fifth-instar larvae (day 3) were dissected and imaged under bright-field microscopy (Olympus, Tokyo, Japan). Pupal weights (day 7) were analyzed using GraphPad Prism 8.4.3 (686).

### 2.7. Scanning Electron Microscopy of Cocoon Silk

Cocoons from WT and *BmPDI*-KO groups were dried, sputter-coated with gold (NeoCoater MP-19020NCTR), and imaged using a Hitachi SU3500 SEM (Hitachi, Tokyo, Japan) at 10 kV and room temperature. Silk fiber diameters are reported as mean ± SD.

### 2.8. Tensile Properties of Cocoon Silk

The diameters of the degummed silk fibers were measured using an optical microscope (Olympus BX51, Olympus, Tokyo, Japan). Then, the degummed silk fibers were mounted on paper frames [27] and tested with a DMA-Q800 analyzer (TA Instruments, New Castle, DE, USA) at 1 mm/min strain rate, 24 °C, and 60% humidity. Breaking strength, strain, Young’s modulus, and toughness were calculated from stress–strain curves (Origin 2021).

### 2.9. Fourier Transform Infrared Spectroscopy

FTIR spectra (Nicolet Nexus, Middleton, WI, USA) of degummed silk were recorded in ATR mode (4000–650 cm^−1^, 0.25 cm^−1^ resolution, 1024 scans). Each spectrum represented the mean of separate deconvolutions for at least 30 separate tests for each sample. The obtained spectra were analyzed using OMSNIC 9 software (Thermo Scientific, Waltham, MA, USA). Amide I deconvolution (PeakFit v4.12) assigned secondary structures of the silk fibers: β-sheet (1610–1628 cm^−1^), α-helix (1648–1660 cm^−1^), random coil (1625–1640 cm^−1^), and β-turn (1660–1700 cm^−1^) [28]. Statistical analysis of the data was performed using the software Origin 2021.

### 2.10. Extraction Analysis of Silk Proteins

Silk samples (20 mg) were dissolved in 500 μL of 9.3 M LiBr (30 min, RT), centrifuged (10,000× *g*, 15 min, 4 °C), and quantified via Bradford assay. Supernatants were stored at –20 °C.

### 2.11. Sample Preparation for LC-MS/MS Detection

Proteolytic hydrolysis: extracted silk cocoon proteins (100 μg) were added to a 0.1% TFA/H_2_O solution (pH 6.5–7.0) with 10 ng/μL of trypsin and incubated at 37 °C for 16 h. Peptide desalination: The hydrolyzed protein samples were filtered by a 10 K ultra-filtration membrane (Millipore, Burlington, MA, USA) and further desalinated using a C18 desalination column (GE, Boston, MA, USA) according to the manuals. Briefly, the C18 desalination column was activated by 200 µL methanol, balanced by 200 µL of 0.1% TFA/ddH_2_O, washed by 200 µL of 0.1% TFA/ddH_2_O, and finally eluted by 200 µL of 80% ACN/0.1% TFA. The eluents were collected and freeze-dried for subsequent use.

### 2.12. LC-MS/MS Analysis

The samples were tested under conditions specified by the manufacturer for LC-MS/MS analysis. Tested conditions: C18 column (3 µm, 100 Å, and 75 µm × 15 cm). Mobile phase A comprised 0.1% formic acid in water, and mobile phase B comprised 0.1% formic acid in ACN. The peptides were eluted by increasing the mobile phase B from 5% to 95% over 60 min. Eluted peptides were analyzed by a high-precision mass spectrometer (Thermo Scientific Q Exactive, Waltham, MA, USA) with a spray voltage of 3.8 kV and a capillary temperature of 320 °C. The parent ion scan range was *m*/*z* 300–1400, the daughter ion scan range was *m*/*z* 100, the AGC of MS1 was 3 × 10^6^, the ion injection time was 60 ms, the AGC of MS2 was 5 × 10^4^, the ion injection time was 80 ms, and the ion screening window was *m*/*z* 3.0. Data were collected in data-dependent mode with a dynamic exclusion of 15 s. The top 20 most abundant precursor ions were selected from a *m*/*z* 100 to 1400 full scan for HCD with a normalized collisional energy of 30%. The resolution of the full MS and MS/MS scans was set at 120,000 and 30,000 on Fusion Lumos and at 70,000 and 17,500 on Q Exactive, respectively. The resulting raw data were compared and analyzed with the *Bombyx mori* protein library using Proteome Discoverer 2.1 software.

### 2.13. Statistical Analysis

Data were analyzed using unpaired Student’s *t*-tests. Significance thresholds: * *p* < 0.05, ** *p* < 0.01, *** *p* < 0.001, **** *p* < 0.0001.

## 3. Results

### 3.1. BmPDI Is Evolutionarily Conserved with the Highest Expression in PSG of Silkworm

**Figure 1:**
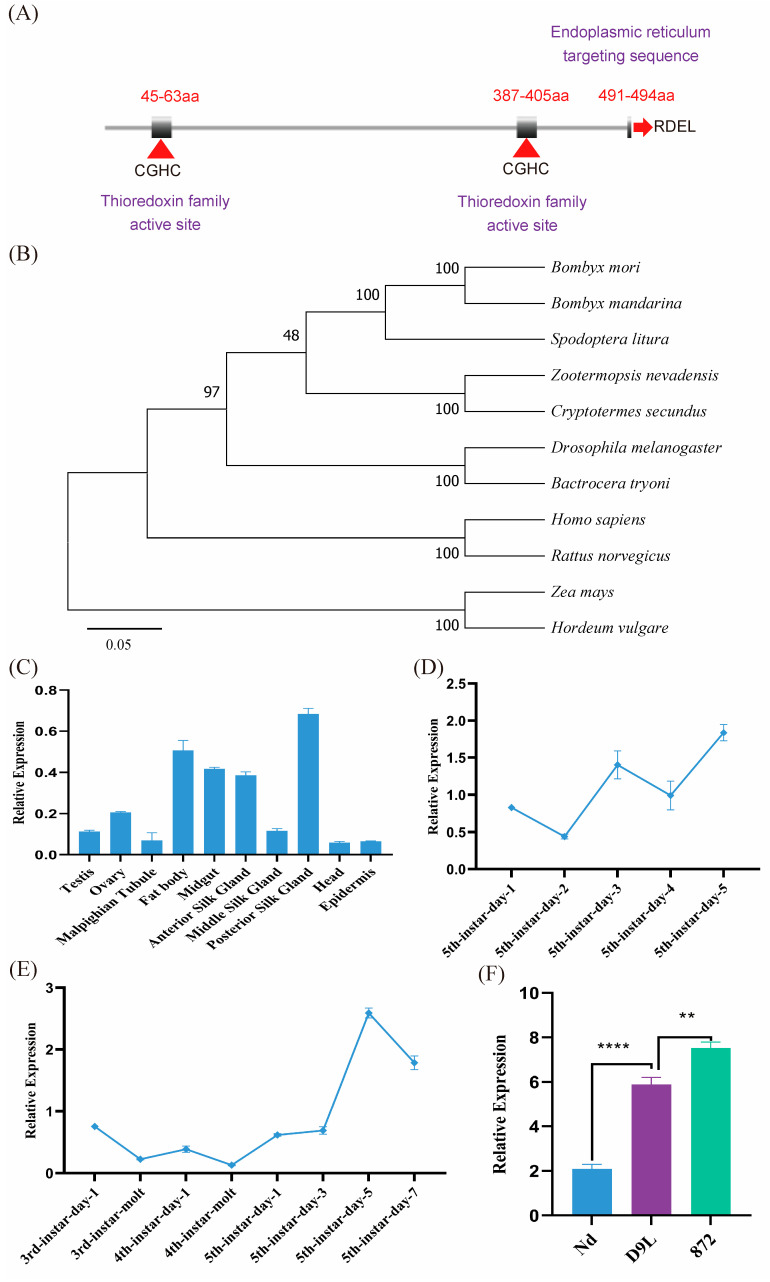
Evolutionary relationship and expression patterns analysis of *BmPDI*. (**A**) Schematic diagram of *BmPDI* structural domain. aa: amino acids. ‘CGHC’ and ‘RDEL’ represent thioredoxin active site sequence and endoplasmic reticulum targeting sequence, respectively. (**B**) Phylogenetic analysis of PDI. (**C**) qRT-PCR analysis of *BmPDI* transcripts in different tissues in the fifth instar on day 3. (**D**) qRT-PCR analysis of *BmPDI* transcripts at different developmental stages. (**E**) qRT-PCR analysis of *BmPDI* transcripts in the posterior silk glands at different developmental stages. (**F**) qRT-PCR analysis of *BmPDI* transcripts in the posterior silk glands in different lines in the fifth instar on day 5. Nd, the cocoon silk mutant strain; D9L, the normal silk-yielding strain; 872, the high silk-yielding strain. **** *p* < 0.0001, ** *p* < 0.01 (Student’s *t*-test).

*BmPDI* possesses a CDS of 1485 bp in length with 10 exons encoding 494 amino acids and contains a signal peptide from a.a. 1 to 17 and two thioredoxin active site sequences at the N- and C-terminals, respectively (Figure 1A). BLAST (https://www.ebi.ac.uk/jdispatcher/, accessed on 15 June 2022) analysis showed that *BmPDI* had 50–99% homology with PDI genes from other species, indicating that PDI is relatively conserved in different species, where functional regions, such as thioredoxin domains and ER retention signals, are highly conserved. The evolutionary analysis showed that *BmPDI* was closely related to that of *Bombyx mandarina* (Figure 1B).

The qRT-PCR results showed that *BmPDI* was widely expressed in various tissues of the fifth instar larvae on day 3, including the posterior silk gland, fat body, midgut, anterior silk glands, ovary, and epidermis, where the highest expression was found in the posterior silk gland (Figure 1C). *BmPDI* expression was higher in the initial stage than in the dormant stage and reached its highest expression on day 5 of the fifth instar (Figure 1D). Furthermore, the expression of *BmPDI* gradually increased in the posterior silk glands of the silkworm from day 1 to 5 of the 5th instar (Figure 1E). Notably, the expression of *BmPDI* considerably varied in different silkworm strains, with relatively low expression in the Nd strain (a naked pupa silkworm strain), approximately 3-fold higher expression in the D9L strain (a normal silk-yielding silkworm strain), and approximately 4-folds higher expression in the 872 strain (a high silk-yielding silkworm strain) (Figure 1F).

### 3.2. CRISPR/Cas9-Mediated BmPDI Knock-Out

**Figure 2:**
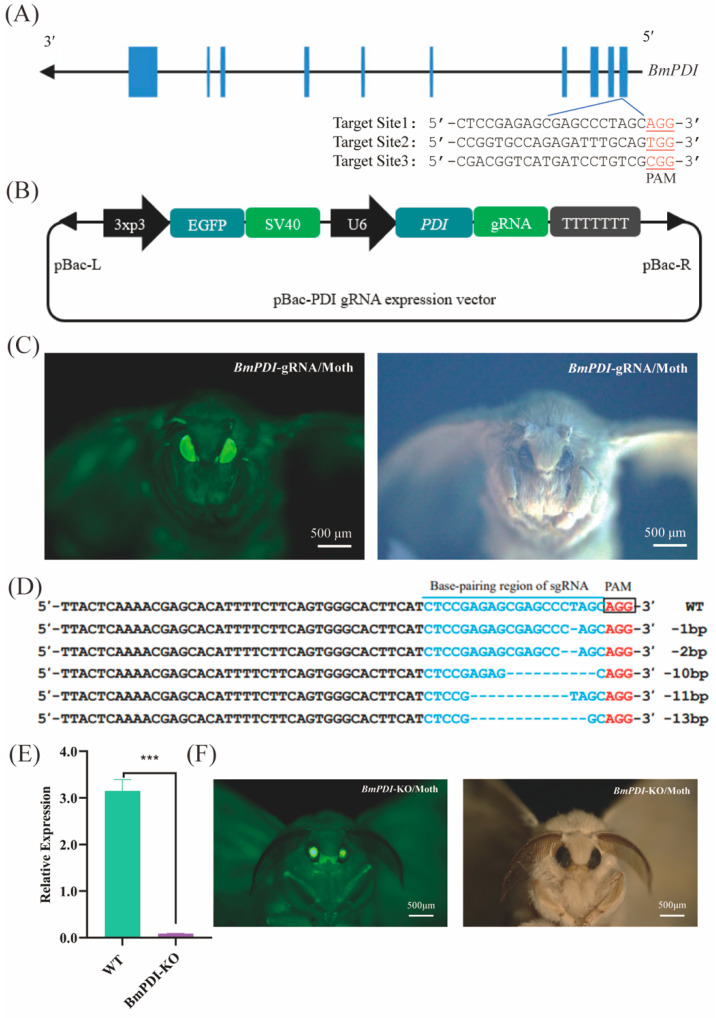
CRISPR/Cas9-mediated *BmPDI* knock-out. (**A**) Schematic diagram of *BmPDI* knock-out target sites design. (**B**) Schematic diagram of the *BmPDI*-gRNA transgenic vector. (**C**) Positive moth individual of the *BmPDI*-gRNA silkworm exposure under green fluorescence light (left) and white light (right). Scale bar is 500 μm. (**D**) Sequence alignment of gRNA-targeted genomic regions. The blue region shows a fragment deletion. (**E**) qRT-PCR analysis of *BmPDI* mRNA. (**F**) *BmPDI* knock-out individuals exposed under green fluorescence light (left) and white light (right). Scale bar is 500 μm. *** *p* < 0.001 (Student’s *t*-test). *BmPDI*, *Bombyx mori* protein disulfide isomerase; WT, wild-type.

To explore the physiological functions of *BmPDI* in silkworms, CRISPR/Cas9-mediated genome editing was conducted to knock out *BmPDI*. Three knock-out target sites were designed in the first exon of *BmPDI* using the CCTop-CRISPR/Cas9 target online predictor (Figure 2A), and the site with the highest prediction score was used to construct the *BmPDI* knock-out gRNA expression vector (Figure 2B). The constructed gRNA expression vector was microinjected into approximately 300 silkworm embryos, and 57 hatched larvae were carefully fed to generate the next progenies; 2 of 20 broods were screened for positive individuals with 3xp3-EGFP expression (Figure 2C, Table 1).

The obtained gRNA-positive individuals were crossed with Cas9*^EXP^*-positive individuals to generate F1 offspring with 3xp3-EGFP and A4-EGFP expression (named *BmPDI*-KO, Figure 2F). Subsequently, genome sequencing of the *BmPDI*-KO individuals showed that deletions of bases or DNA fragments occurred with an editing efficiency of approximately 55% in the genomes of the silk gland tissues of the *BmPDI*-KO individuals (Figure 2D). The mRNA expression level of *BmPDI* was significantly reduced in *BmPDI*-KO individuals (Figure 2E). These results indicated that *BmPDI* was successfully knocked down in the *BmPDI*-KO strain.

### 3.3. Deletion of BmPDI Inhibits the Development of Silk Glands and Decreases the Silk Yields

**Figure 3:**
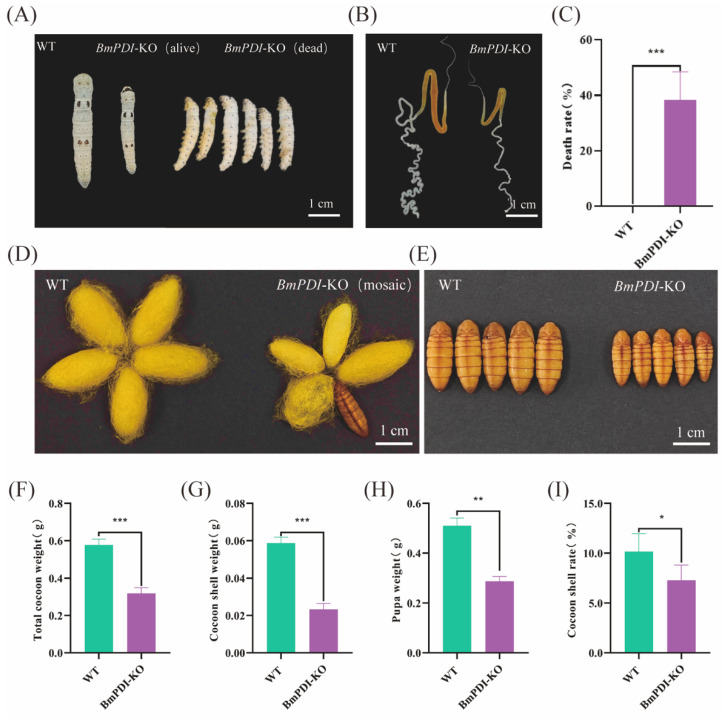
Knock-out of *BmPDI* inhibits silk gland development and reduces silk yields. (**A**) Phenotypes of silkworm larvae on day 3 of the fifth instar from the WT and *BmPDI*-KO silkworms. (**B**) Phenotypes of the silk gland on day 7 of the fifth instar from the WT and *BmPDI*-KO silkworms. (**C**) Death rate analysis of silkworm larvae between WT and *BmPDI*-KO silkworms. (**D**,**E**) Phenotypic observation of cocoon and pupa. (**F**–**I**) Statistical analysis of economic traits after *BmPDI* knock-out, including the total cocoon weight, cocoon shell weight, pupa weight, and cocoon shell rate. *** *p* < 0.001, ** *p* < 0.01, * *p* < 0.05 (Student’s *t*-test). *BmPDI*, *Bombyx mori* protein disulfide isomerase; WT, wild-type; *BmPDI*-KO, *BmPDI*-knockout.

During feeding of the *BmPDI*-KO and WT silkworms, some individuals in the *BmPDI*-KO group exhibited significant phenotypes of smaller body size, delayed development, approximately 38.46% lethality during the larval stage, and smaller silk glands than WT silkworms (Figure 3A–C). Some of the *BmPDI*-KO individuals that successfully developed to the silk-spinning stage produced smaller cocoons with thinner cocoon shells, failed to spin silk, and finally became naked pupae (Figure 3D,E). The total cocoon weight, cocoon shell weight, pupal weight, and cocoon shell rate of the *BmPDI*-KO individuals were statistically analyzed and showed a significant decrease compared to the WT individuals (Figure 3F–I), indicating that knockout of the *BmPDI* significantly affected the silk yields of silkworms.

### 3.4. Deletion of BmPDI Reduces the β-Sheet Crystal of Silk and Decreases the Silk’s Mechanical Properties

**Figure 4:**
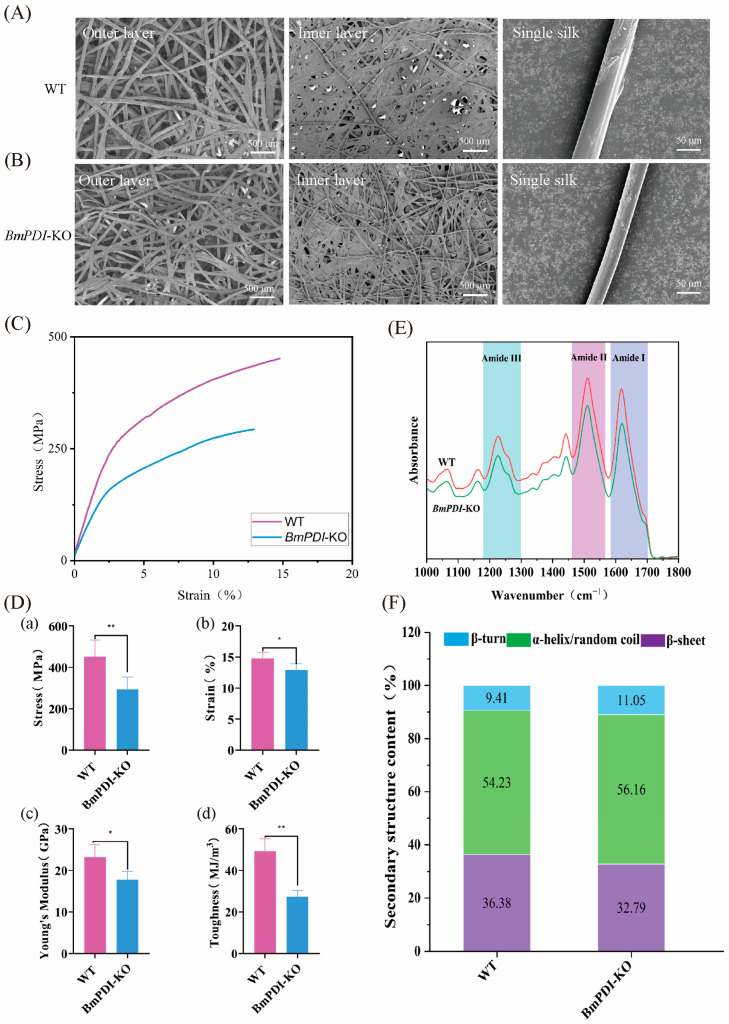
Morphology and the mechanical property analysis of silk fibers between WT and *BmPDI*-KO silkworms. SEM micrographs of the outer and inner layers of the cocoon and single silk fiber of WT (**A**) and *BmPDI*-KO (**B**), respectively. (**C**) Stress–strain curves of WT and *BmPDI*-KO raw silk fibers. (**D**) Calculation of the breaking strength (a), breaking strain (b), Young’s modulus (c), and toughness (d) of WT and BmPDI-KO raw silk fibers. (**E**) ATR-FTIR spectra from 1000 to 1800 cm^−1^ of WT and BmPDI-KO raw silk fibers. (**F**) β-sheet content of WT and *BmPDI*-KO raw silk fibers. ** *p* < 0.01, * *p* < 0.05 (Student’s *t*-test). *BmPDI*, *Bombyx mori* protein disulfide isomerase; WT, wild-type; *BmPDI*-KO, *BmPDI*-knockout.

We further analyzed the effect of *BmPDI* knock-out on the microstructure of the cocoons. The morphologies of the outer surface, inner surface, and single silk fibers of the *BmPDI*-KO cocoons, which were sparser, thinner, and finer than those of the WT cocoons, were observed by SEM (Figure 4A,B). The average diameter of a single silk fiber from the *BmPDI*-KO strain was approximately 14.55 μm, which decreased by 23.9% compared to the approximately 19.13 μm of the WT strain. Subsequently, the mechanical properties of silk from the WT and *BmPDI*-KO cocoons were compared, which showed that the mechanical properties of the *BmPDI*-KO silk were significantly lower than those of the WT silk (Figure 4C); the breaking strength, breaking strain, Young’s modulus, and toughness decreased by 35.10%, 12.59%, 23.44%, and 44.44%, respectively (Figure 4E), suggesting that the deletion of *BmPDI* affected the mechanical properties of silk.

In general, the secondary structural conditions of silk fiber were related to its mechanical properties, which determined the reason for the decrease in the mechanical properties of silk in the *BmPDI*-KO silkworm. Fourier transform infrared spectroscopy analysis showed that three typical absorption peaks at 1700–1600, 1600–1500, and 1300–1200 cm^−1^, each representing amide I, amide II, and amide III of the silk [29,30], respectively, were found in the infrared spectrum (Figure 4D), which were used to calculate the secondary structural content of silk. In addition, a deconvolution analysis of the amide I bands was conducted to calculate the secondary structural content of the silk (Figure S2), which showed that the β-sheet crystal content in the *BmPDI*-KO silk significantly decreased compared to that of the WT silk (Figure 4F). *Bombyx mori* silk is a semi-crystalline biopolymer with highly ordered inverted parallel β-sheet crystals embedded in an amorphous matrix. The β-sheet crystals are the physical cross-linking points of the molecular network inside the silk, which considerably influence the physical properties of the silk [29,31,32,33]. Silk with a higher β-sheet structure typically exhibits considerable strength. Consequently, this result indicated that the decrease in β-sheet structure content might be one of the reasons for the decrease in mechanical properties of *BmPDI*-KO silk.

### 3.5. BmPDI Knock-Out Decreases the Silk Yields and Mechanical Properties by Regulating Post-Translational Modification of Silk Proteins

**Figure 5:**
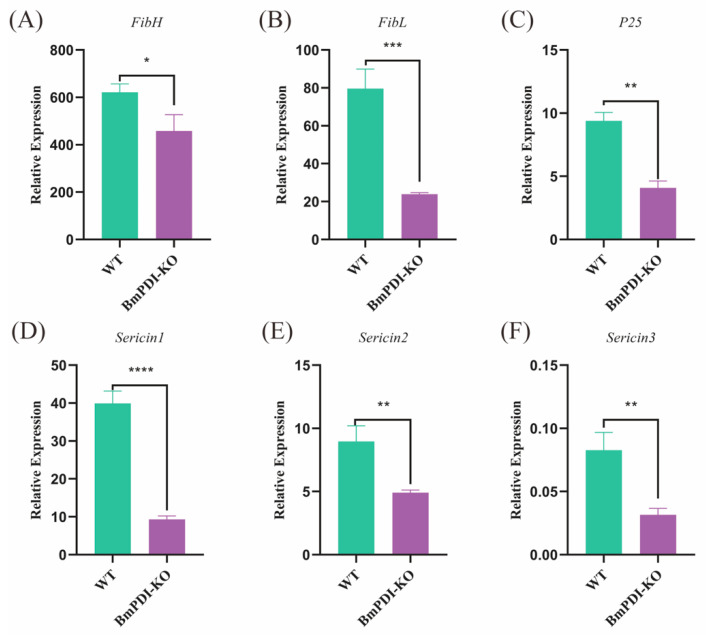
Transcriptional level analysis of silk protein synthesis-related genes. (**A**–**C**) Detection of expression levels of genes related to fibroin proteins. (**D**–**F**) Detection of expression levels of genes related to Sericin proteins. **** *p* < 0.0001, *** *p* < 0.001, ** *p* < 0.01, * *p* < 0.05 (Student’s *t*-test).

To further understand the effects of *BmPDI* knock-out on the synthesis or secretion of silk proteins, the expressions of silk protein-related genes were detected using qRT-PCR. The expressions of fibroin- and sericin-related genes were significantly downregulated at the mRNA level, among which *FibH*, *FibL*, and *P25* were downregulated 1.4, 3.3, and 2.3 times, respectively (Figure 5A–C), and *Sericin1*, *Sericin2*, and *Sericin3* were downregulated 4.3, 1.8, and 2.6 times, respectively (Figure 5D–F), implying that the deletion of *BmPDI* gene affected the synthesis or secretion of silk proteins.

**Figure 6:**
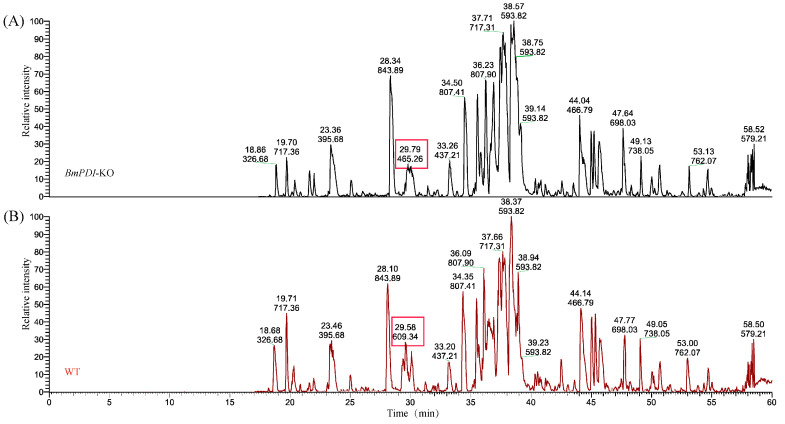
LC-MS/MS spectra to detect disulfide bonds of silk proteins from WT and *BmPDI*-KO silkworms. (**A**) LC-MS/MS spectrum of silk proteins from the *BmPDI*-KO silkworm. (**B**) LC-MS/MS spectrum of silk proteins from the WT silkworm. Red boxes represent that the peak times and intensities significantly differ between WT and *BmPDI*-KO strains. WT, wild-type; *BmPDI*-KO, *BmPDI*-knockout.

PDI is a highly abundant protein in the ER that acts as a molecular chaperone, aiding and facilitating the formation of disulfide bonds and proper folding and assembly of proteins [34]. We speculated that deletion of the *BmPDI* might affect the formation of disulfide bonds in silk proteins, affecting their processing in the ER. Subsequently, the disulfide bond content in the major silk proteins of the *BmPDI*-KO silk was examined and analyzed using LC-MS/MS (Figure 6). The number of disulfide bonds involved in FibH, FibL, and P25 proteins was significantly reduced in *BmPDI*-KO silkworm silk compared to that in WT silkworm silk (Table 2), suggesting that *BmPDI* might regulate the formation of disulfide bonds in silk proteins through post-translational modification.

**Figure 7:**
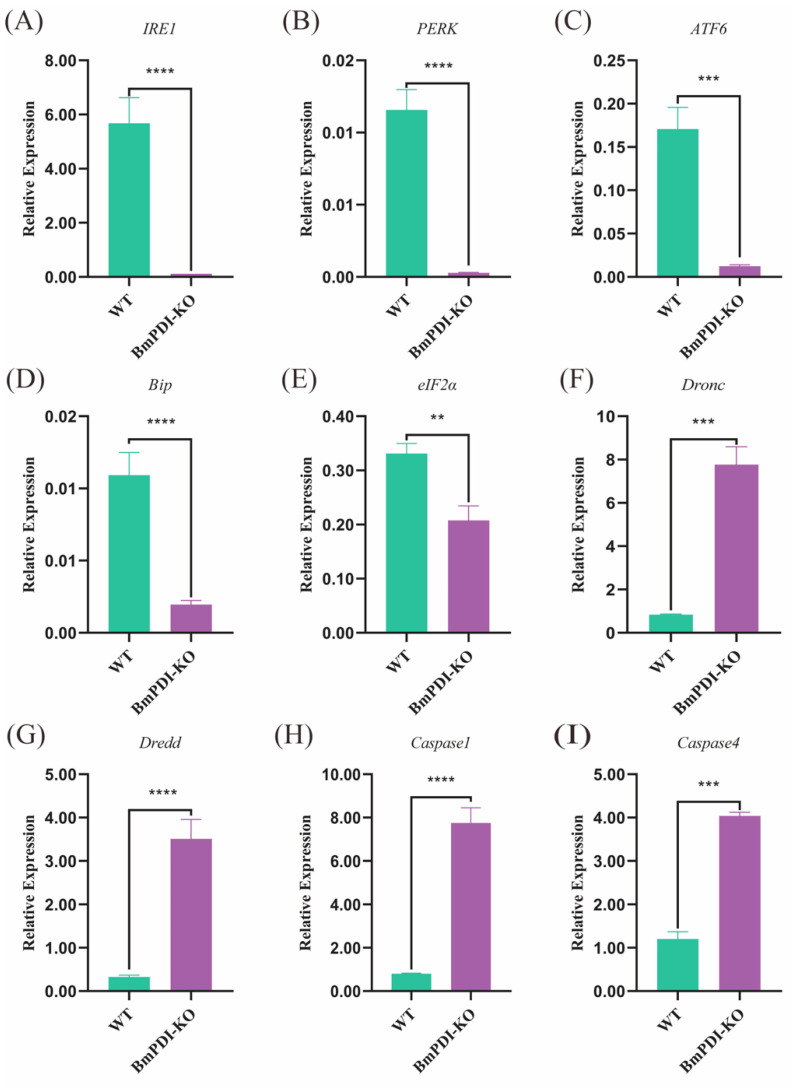
Transcriptional level analysis of the UPR and apoptosis pathway-related genes. (**A**–**E**) Detection of expression levels of the UPR signaling pathway-related genes. (**F**–**I**) Detection of gene expression levels associated with apoptosis. **** *p* < 0.0001, *** *p* < 0.001, ** *p* < 0.01 (Student’s *t*-test).

PDI is closely related to the unfolded protein response (UPR) in ER stress response [35]. Subsequently, the expressions of the UPR and apoptosis pathway-related genes were detected using qRT-PCR. The transcript levels of the UPR pathway-related genes were significantly downregulated in the *BmPDI*-KO silkworm; *IRE1*, *PERK*, and *ATF6* were downregulated 351, 41, and 13 times, respectively, whereas *Bip* and *eIF2α* were downregulated 5 and 1 times, respectively (Figure 7A–E). However, some key apoptotic pathway genes, such as *Dronc*, *Dredd*, *Caspase1*, and *Caspase4*, were significantly upregulated 8, 10, 9, and 2 times, respectively (Figure 7F–I). These results suggested that deletion of the *BmPDI* gene severely suppressed the expression of UPR pathway-related genes and substantially promoted the expression of apoptotic pathway-related genes, which accelerated the apoptosis of silk gland cells and inhibited silk gland growth and development.

## 4. Discussion

In this study, *BmPDI* was systematically analyzed for its evolutionary conservation and high expression patterns in multiple silkworm tissues. The *BmPDI*-KO silkworm was generated by CRISPR/Cas9-mediated gene editing with significant phenotypes of smaller body, silk gland, and cocoon sizes; thinner silk fibers; and significantly reduced silk yields and mechanical properties compared to the WT silkworms.

The *Fib-H*, *Fib-L*, and P25 glycoproteins formed a basic unit of silk fibroin fibers with a ratio of 6:6:1 in *Bombyx mori* [3]. The H- and L-chain were connected to an H-L-chain subunit by a disulfide bond between Cys-20 of *Fib-H* and Cys-172 of *Fib-L* [5], which benefited both intracellular transport of fibroin (migration from the rough ER region to the Golgi apparatus) and secretion into the glandular cavity [36,37]. The *Fib-L* of the silkworm strains Nd-s and Nd-s^D^ undergo mutations that prevent the formation of disulfide bonds with the *Fib-H* of fibroin, resulting in an exposed free thiol group in the fibrillar H-chain, hindering the transport of the fibroin L-chain from the ER to the Golgi apparatus, resulting in a secretion level of fibroin proteins that is only 1% of that of normal silkworms [36]. PDI can mediate the formation and rearrangement of disulfide bonds in proteins [17] and acts as a molecular chaperone to facilitate correct protein folding in the ER [34]. This study showed that *BmPDI* exhibited high expression levels in the posterior silk glands of silkworms, with gradually increasing expression levels in the posterior silk glands during the fifth larval instar when abundant silk proteins were synthesized. Thus, we speculate that *BmPDI* may have affected the synthesis or secretion of silk proteins during this period. In addition, this study highlighted that the deletion of *BmPDI* in silkworms caused significant reductions in silk yield and transcription levels of silk protein genes, further proving that the loss-of-function of *BmPDI* could affect the synthesis and secretion of silk proteins.

*Bombyx mori* silk is a semi-crystalline biopolymer with highly ordered inverted parallel-sheet crystals embedded in an amorphous matrix. The β-sheet crystals are generally regarded as the physical cross-linking points of the molecular network inside the silk and the main factor affecting the physical properties of the silk [29,31,33,38]. Therefore, the mechanical properties of silk were closely related to the β-sheet crystals content in silk [27,38,39,40]. In this study, knocked-out *BmPDI* decreased the secondary structure of silk β-sheet content, significantly reducing silk mechanical properties. This further indicates the close correlation between the β-sheet content and the mechanical properties of silk.

The ER is the primary site of protein synthesis in eukaryotic cells, where numerous proteins are processed. In silkworms, the fibroin protein is first folded and assembled in the ER of posterior silk gland cells. This process involves the formation of disulfide bonds, which help the silk protein form a stable structure, followed by translocation to the Golgi apparatus, where it is secreted out of the cell. PDI is a highly abundant protein in the ER, whose primary function is to act as a molecular chaperone, aiding and facilitating the formation of disulfide bonds and proper folding and assembly of proteins [34]. In this study, deletion of the *BmPDI* significantly reduced the number of disulfide bonds involved in the FibH, FibL, and P25 proteins in silkworm silk, further proving that *BmPDI* might severely affect the processing of silk proteins. Furthermore, this study showed that deletion of the *BmPDI* also caused significant transcriptional downregulation of UPR signal pathway-related genes and upregulation of apoptotic pathway-related genes in the *BmPDI*-KO line, which implied that *BmPDI* regulated the growth and development of silkworm larvae and silk glands by participating in the UPR signal in *Bombyx mori*.

## 5. Conclusions

This study demonstrated the crucial role of *BmPDI* knock-out in regulating the post-translational modification of silk proteins, which affected the disulfide bond formation, synthesis, yield, and mechanical properties of the silk protein, as well as the development of the silk gland and silkworm larvae through the UPR and apoptosis pathways. Therefore, this study provides a potential target gene for modifying the silk yield and mechanical properties of *Bombyx mori* in the future.

## Figures and Tables

**Table 1:** Summary of microinjection information for *Bombyx mori*.

*Bombyx mori* Line	G0 Eggs Microinjected	G0 Eggs Hatched	G1 Moth Broods	Positive G1 Moth Broods	Positive Ratio
D9L	300	57	20	2	10%

**Table 2:** Number of disulfide bonds in silk proteins of two silkworm strains.

Category Name	Number of Disulfide-Linked Peptides in WT Lines	Number of Disulfide-Linked Peptides in *BmPDI*-KO Lines
FibH protein	1	0
FibL protein	56	41
P25 protein	70	53
Total	127	94

## Data Availability

The original contributions presented in this study are included in the article/Supplementary Materials. Further inquiries can be directed to the corresponding author.

## Supplementary Materials

The following supporting information can be downloaded at: https://www.mdpi.com/article/10.3390/insects16070684/s1, Figure S1: Statistics of average diameter of cocoon silk in WT and *BmPDI*-KO; Figure S2: FTIR analysis of silk fiber of each silkworm strains. (A) Deconvolution of amide I band of FTIR spectrum in WT; (B) Deconvolution of amide I band of FTIR spectrum in *BmPDI*-KO; Table S1: Primers for qRT-PCR detection.

## Abbreviations

ASG	Anterior silk gland
MSG	Middle silk gland
PSG	Posterior silk gland
Nd	Naked pupa
qRT-PCR	Real-time quantitative-PCR
FibH	Fibroin heavy protein
FibL	Fibroin light protein
P25	Protein 25 kDa
Ser1	Sericin-1
Ser2	Sericin-2
Ser3	Sericin-3
PDI	Protein disulfide isomerase
ER	Endoplasmic reticulum
ERS	Endoplasmic reticulum stress
UPR	Unfolded protein response
Bip	Immunoglobulin-binding protein
IRE1	Inositol-requiring protein 1
ATF6	Activating transcription factor 6
PERK	Protein kinase RNA-like ER kinase
eIF2α	eukaryotic translation initiation 2
gRNA	guide RNA
PAM	Protospacer-associated motif
CDS	Coding sequence
WT	Wild type
aa	Amino acids
Dronc	Drosophila Nedd2-like caspase
Dredd	Death related ced-3/Nedd2-like protein
