# Supplementary material for: Knocked-Out Bombyx mori Protein Disulfide Isomerase Decreases Silk Yields and Mechanical Properties by Affecting the Post-Translational Modification of Silk Proteins
Source: Insects. 2025 Jun 30;16(7):684. doi: 10.3390/insects16070684 (PMC12295915; doi:10.3390/insects16070684)
Supplement: Supplementary file 1 [file insects-16-00684-s001.zip › insects-3698503-supplementary.pdf]

Supplementary Information

ORIGINAL ARTICLE

Knocked-out Bombyx mori protein disulfide isomerase decreases silk yields and mechanical properties by affecting the post-translational modification of silk proteins

Shifeng Yang[1], Mengyao He[1], Xian Li[1], Huan Dong[1], Hexu Lei[1], Fangyu Wang[1], Hanxin Deng[1], Hongji Zhou[1], Siyu Chen[1], Yujuan Zhou[1], Zihan Meng[1], Ding Tu[1], He Wang[1], Qingyou Xia[1], Feng Wang[1*]

[1] Integrative Science Center of Germplasm Creation in Western China (CHONGQING) Science City, Biological Science Research Center, Southwest University, Chongqing 400715, China.

Correspondence: Feng Wang, Integrative Science Center of Germplasm Creation in Western China (CHONGQING) Science City, Biological Science Research Center, Southwest University, Chongqing 400715, China. Tel: +86 23 68251987; Fax: +86 23 68251128; email: wangf1986@swu.edu.cn

Table S1 Primers for qRT-PCR detection

Primer name	F primer （5′ - 3′）	R primer （5′ -3′）
sw22934	TTCGTACTGCTCTTCTCG	CAAAGTTGATAGCAATTCCCT
BmPDI	CTAGCGAAAGTTGACGCAACTC	TGCATAGGACTGCCATTCCTG
Fib-H	TCTGTGTCATCTGCTTCATCTCG	TATCCAGGACGAAGTAAGAAAC
Fib-L	ATACCGATTGGTCACATAACAG	GCAGATAGATGGGCGATAA
P25	AGCCGCTGTGGCAGTTTTG	TAGGTGGCGTTGAAGTATGG
Sericin1	CACAACCGATAAGACGAG	GACGAAGTGGAGGAAGC

Sericin2	CATCGGCTGACTACCA	AGAGTTGCTGCCCTTAC
Sericin3	TGTCTCGTCGGTGGAA	TTGTTGTATGACTGGCTCT
ATF6	GAGTCGTTGGATTAGAGGAGGC	GATGTTACGCACCTGATTTCTTG
Bip	CCGCCCTTTAACTTTCCACTC	GACACGCTGCCGTCGCTA
eIF2α	CATCACAGAGGCAGGTGGAGT	CAGCGAGTTCAGCGTTTTCA
PERK	TGGCTTTGGCGTTAGTCTTGTT	CTCGGATGGTATGTCCTCGTT
IRE1	GTTGGGCTGCGTGTTCTATTAC	AGAACATCGGGTATTTCAGTATCG
Dronc	TGTGGCTGTCTTCCTTC	ATCTAAGTCTGTGCCCTC
Dredd	AGCCGTATGACATGGTATCT	TGTTTCCAAGTGGGTTTC
Caspase1	CTTCACTGCTGATAAATGTCC	TTTCTCCAAGAGTAATAACCTG
Caspase4	GAAATACGCTACGACATACG	ACGACTTCAAAGCCAAACT

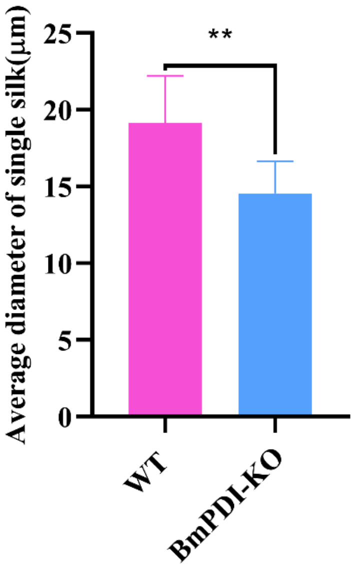

Figure S1. Statistics of average diameter of cocoon silk in WT and *BmPDI*-KO. ** $p < 0.01$ (Student's *t*-test)

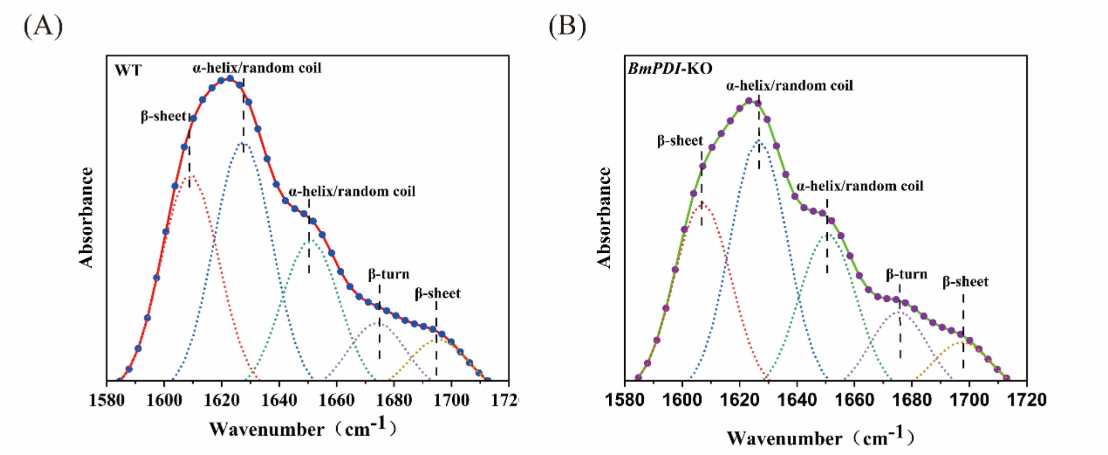

Figure S2. FTIR analysis of silk fiber of each silkworm strains. (A) Deconvolution of amide I band of FTIR spectrum in WT; (B) Deconvolution of amide I band of FTIR spectrum in *BmPDI*-KO.
